# Investigating the Electrochemical Double Layer with Quantum‐Chemical Simulations and Implicit Solvation Models

**DOI:** 10.1002/chem.71502

**Published:** 2026-08-11

**Authors:** Alessandro Mangiameli, Christopher J. Stein

**Affiliations:** ^1^ Department of Chemistry and Catalysis Research Center TUM School of Natural Sciences Technical University Munich Garching Germany; ^2^ Atomistic Modeling Center Munich Data Science Institute Garching Germany

**Keywords:** electric double layer, implicit electrolyte, Poisson–Boltzmann, RISM

## Abstract

We assess the dielectrically consistent reference interaction site model (DRISM) as an implicit electrolyte framework for modeling the electrochemical double layer, and compare it with the Poisson–Boltzmann model and explicit molecular dynamics results from the literature. We use the gold–electrolyte interface as the main test case and analyze solvent and ionic density profiles, the differential capacitance, and the solvation contribution to CO adsorption. The results show a strong sensitivity to the Lennard‐Jones parametrization of metal–ion and metal–water interactions. In particular, we find that the default Lorentz–Berthelot mixing rules are inadequate and lead to excessive 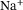 accumulation at the interface, which results in an increase of the differential capacitance at negative electrode potentials. We demonstrate that introducing pair‐specific metal–ion parameters yields more symmetric charging behavior and provides greater flexibility. Our findings suggest that using pair‐specific parameters, rather than relying on Lorentz–Berthelot mixing rules, improves the accuracy of the model and opens the way for future studies with this improved yet equally performant model.

## Graphical abstract

ESM‐RISM is applied to model electrochemical interfaces and benchmarked against Poisson–Boltzmann and molecular dynamics results. Density profiles, differential capacitance, and adsorption energetics at the gold–electrolyte interface are analyzed, highlighting the capabilities of this implicit electrolyte framework.

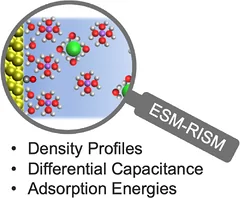

**Abbreviations**
EDL: Electrical double layer
PB: Poisson–Boltzmann
LPB: Linear Poisson–Boltzmann
NLPB: Non linear Poisson–Boltzmann
CANDLE: Charge‐asymmetric nonlocally determined local electric response
AIMD: Ab initio molecular dynamics
DFT: Density functional theory
QM: Quantum mechanics
MM: Molecular mechanics
MD: Molecular dynamics
RISM: Reference interaction site model
XRISM: Extended RISM
DRISM: Dielectrically consistent RISM
ESM: Effective screening medium
LJ: Lennard‐Jones
LB: Lorenz–Berthelot
UFF: Universal force field
KH: Kovalenko–Hirata
IHP: Inner Helmholtz plane
OHP: Outer Helmholtz plane
PZC: Potential of zero charge

## 1 Introduction

The structure and properties of the Electrical double layer (EDL) at charged interfaces play a central role in electrochemistry. From energy storage in supercapacitors to electrocatalysis and corrosion, the interfacial region controls ion distributions, potential profiles, and ultimately the rates and selectivity of electrochemical processes [1, 2, 3]. A quantitative understanding of the EDL is therefore essential for the design of efficient electrochemical systems.

Atomistic simulations of interfacial phenomena must adequately model the intricate interplay among solvent, ions, and charged surfaces. The most common theoretical framework for EDL is the Poisson–Boltzmann (PB) model originally developed by Gouy [4] and Chapman [5] and later by Stern [6]. Its mean‐field formulation, in which ions are treated as point charges in a continuum dielectric, allows for simple, computationally inexpensive evaluation of ion density profiles and potential distributions. Thanks to its simplicity, the PB model (and related models [7]) is, even today, one of the most common approaches, and it is implemented in various well‐known electronic structure software packages [8, 9, 10, 11, 12]. Nevertheless, its approximations neglect molecular granularity and correlation effects, which become critical at high ionic strength or in the immediate vicinity of electrodes.

A much more detailed picture can be obtained from first‐principles methods like Ab initio molecular dynamics (AIMD), where the electrolyte is modeled explicitly at the Density functional theory (DFT) level [13, 14, 15, 16]. AIMD is, in principle, providing the most accurate description; however, it is computationally very demanding and therefore limits the size of the simulation box and the time scale of the simulation. To reduce the computational cost, the electrolyte is often treated classically in a Quantum mechanics (QM)/Molecular mechanics (MM) framework [3]. However, even with this substantially decreased computational cost, sufficient sampling is challenging since long equilibration times and large simulation cells are usually required [17]. This becomes especially challenging in constant‐potential simulations, where specialized algorithms to control the electrode potential while maintaining charge neutrality need to be applied [17].

An alternative approach is provided by treating the electrolyte with the Reference interaction site model (RISM). RISM, first introduced by Chandler and Andersen [18], is an approximation to solve the molecular Ornstein–Zernike equation and obtain the site–site radial distributions functions for the given system; it has been extended to molecular fluids with charged sites by Hirata and Rossky [19] also indicated as Extended RISM (XRISM) and then further developed for electrolytes by Perkyns [20, 21], who introduced a dielectrically consistent variant which apport a correction to accounts for the solvent polarizability. RISM has been first embedded to QM calculations by Sato et al. [22] and further extended for electrochemical simulations by Nishihara and Otani [23] and combined with the Effective screening medium (ESM) [24], in short ESM‐RISM, which effectively removes the periodicity in the direction perpendicular to the slab, enabling a non‐repeated vacuum/slab/solvent configuration. The QM/ESM‐RISM framework has been employed in various studies [25, 26, 27, 28] and proved to yield essential insights into the structure of the electrode–electrolyte interface. In two recent works, ESM‐RISM has been compared to the linearized PB model for studying of the CO reduction reaction at the Cu(100) facet [29] and with the Charge‐asymmetric nonlocally determined local electric response (CANDLE) model [9] (which also belongs to the family of the linearized PM models) for the studying of the standard hydrogen electrode [30]. Both studies report that ESM‐RISM generally leads to destabilization of the adsorption energies within the employed parametrizations. In the second study, Demeyere and Skylaris compared the differential capacitance computed with CANDLE and ESM‐RISM, noticing considerable differences between the two models.

In this work, we extend the previous studies by further evaluating the capabilities of QM/ESM‐RISM and of the newly developed dielectrically consistent version Dielectrically consistent RISM (DRISM) [31], for the modeling of the electrode‐electrolyte interface. In the first part, we focus on the solvent and ionic density profiles at an Au(111) facet. In the second part, we study the differential capacitance and its dependence on the electrolyte parametrization, compositions, and concentrations, since this quantity can be compared rather directly to experimental studies. Finally, we test the impact of the parametrization for the adsorption energy of CO at the Au surface. We systematically compare the DRISM results with the recently implemented nonlinear PB model VASPsol++ [32].

## 2 Results and Discussion

### 2.1 Density Profiles

The most direct outputs of a QM/DRISM calculation are the classical particle density distributions for the implicit DRISM electrolyte around the explicit QM system. The solvent and ionic density profiles are the x‐ and y‐averaged particle densities as a function of the slab distance z. Since these profiles cannot be directly measured experimentally, computational models such as DRISM are valuable tools for elucidating the structure of the EDL.

#### 2.1.1 Solvent Density Profiles

The structure of the metal–water interface has been extensively investigated. For non‐absorbing metals like Au, water is known to form a layer in which the water molecules lie parallel to the surface [13, 33, 34]. For adsorbing metals like Pt, the peak for the oxygen appears split, due to its specific adsorption on the surface [13]. As already reported by Demeyere and Skylaris [30], QM/RISM is unable to predict the double‐peak structure, given the lack of specific chemical bonding interactions in the metal–water potential, and we would expect the same deficiency in a fully classical Molecular dynamics (MD) or a QM/MM simulation, where the electrolyte is modeled classically.

To validate the water density profiles predicted by DRISM for the less reactive Au(111) facet, we compare them with the particle and charge profiles obtained from the classical MD simulations of Hughes and Walsh [35] (Figure 1).

**FIGURE 1:**
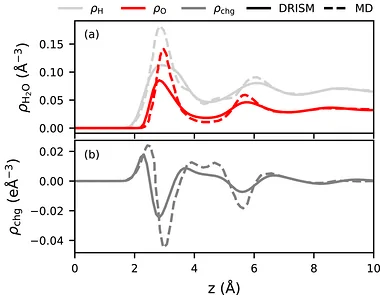
Panel (a) displays hydrogen (light gray) and oxygen (red) particle density profiles computed with DRISM (continuous line) and explicit MD simulations (segmented line) [taken from Hughes et al. [35]] for the Au(111) facet in water. The mTIP3P water model is used in both cases. For the DRISM calculation, we adjusted the LJ parameter for Au to match the Au–O GolP parametrization. Panel (b) shows the respective charge density profiles.

The mTIP3P water model is used in both cases. We highlight, however, that an exact correspondence is not expected, since the classical MD [35] is performed with the GolP‐CHARMM force field, which uses dipole sites on the gold surface to simulate the metal polarization, while in the QM/DRISM embedding, the polarization is taken into account directly by the density functional. For the hydrogen and oxygen density profiles, the positions of the first and second peaks are in good agreement; however, the height of the peaks predicted by DRISM is lower; this tendency might also be ascribed to the Kovalenko–Hirata (KH) closure, which has been reported to underestimate the height of the correlation peaks [36]. As a consequence, the peaks in the charge density profiles are also lower in DRISM than in MD. Despite these differences, we observe a generally good agreement between DRISM and the classical MD simulations.

For completeness, we also mention that, while for DRISM and classical MDs, there is a one‐to‐one mapping between the particle density profiles and the charge density profiles, this is not the case for AIMD, where the electronic charge is obtained from DFT; therefore, even for similar particle density profiles, the charge density profiles might be slightly different.

When a potential is applied to the electrode, water molecules are tilted by electrostatic interactions, resulting in a shift in the hydrogen and oxygen peaks. This behavior is observed in both classical [37] and ab initio calculations [14] as well as in QM/MM [3], and has already been documented for QM/RISM [30]. However, a quantitative comparison between the QM/RISM and MD density profiles at finite applied potential is more complicated due to two reasons: first the different definitions of the electrode potential in the two methods, which is based on the Fermi level referenced to the electrolyte bulk potential in QM/RISM and on the surface charge in classical MD, respectively, and second the important role of their distinct treatments of metal polarization.

We now turn to the comparison between DRISM and the PB model. In Figure 2, we show a side‐by‐side comparison of the two models for both negative $\sigma =-8.3\;\mu C/{\text{cm}}^{2}$ and positive $\sigma =+8.3\;\mu C/{\text{cm}}^{2}$ surface electrode charge, which correspond to a negative and positive applied potential relative to the Potential of zero charge (PZC).

**FIGURE 2:**
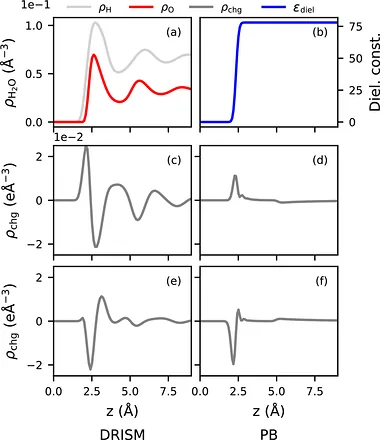
Comparison between solvent modeling in PB and DRISM. In (a), the particle density profiles of water computed by DRISM are shown, whereas in the PB model, the solvent is replaced with a continuum dielectric. In (c) and (d) are shown the charge‐density profile from DRISM and the bound charge from the PB for a negative electrode surface charge $\sigma =-8.3\;\mu C/{\text{cm}}^{2}$ potential with respect to the PZC. In (e) and (f), the same applies for a positive electrode potential.

In the PB model, the solvent is modeled as a dielectric continuum, as shown in panel (b). In the presence of the solute, the dielectric medium generates a polarization field P(r); the bound (or polarization) charge is then defined as $\rho_{b}\left(r\right)=-\nabla P\left(r\right)$. As we can see, while the charge profile predicted by DRISM exhibits complex oscillatory behavior, the bound charge obtained from the PB model shows mainly one peak situated at the boundary of the dielectric cavity, followed by an exponential peak due to explicit ionic charge screening.

#### 2.1.2 Ionic Density Profiles

We now move our attention to the ionic density profiles. A central step in the QM/RISM framework is selecting the pair‐potential parameters. In the literature, numerous Lennard‐Jones (LJ) parametrizations for water–ion systems have been proposed, each optimized for different thermodynamic or structural properties [38, 39, 40]. We discuss the Au(111) facet as a common catalytic surface in contact with aqueous NaCl as a typically used electrolyte solvent. In Figure 3, we present the ionic density profiles for an electrolyte concentration of 1M, obtained (from top to bottom) at negative, neutral, and positive surface charge, respectively.

**FIGURE 3:**
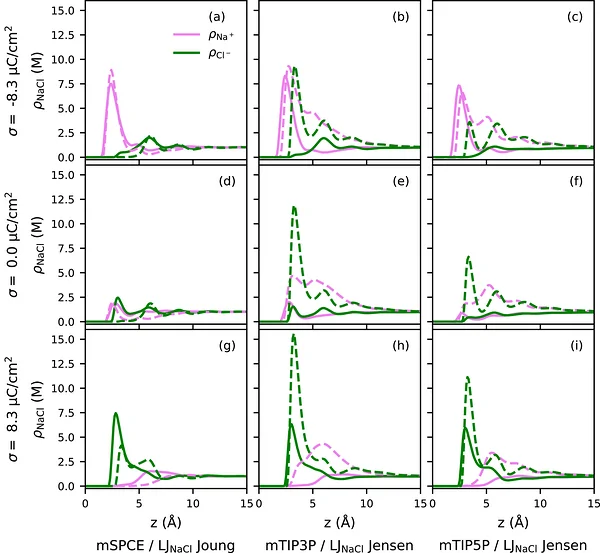
Ionic density profiles for the Au(111) facet in contact with 1M NaCl. (a–c) negative surface charge $\sigma =-8.3\;\mu C/{\text{cm}}^{2}$; (d–f) neutral electrode $\sigma =0\;\mu C/{\text{cm}}^{2}$; (g–i) $\sigma =+8.3\;\mu C/{\text{cm}}^{2}$. The continuous lines refer to the UFF parametrization for the Au electrode, while the segmented line refers to the parametrization from Heinz et al. [41].

For the electrolyte, we use three different water/ions parametrizations: the mSPCE water model with the ionic parametrization from Joung and Cheatham [38] (left), the mTIP3P water model with the ionic parametrization from ensen and Jorgensen [39] (middle), and the mTIP5P water model, again with the parametrization from Jensen and Jorgensen [39] (right). For the gold surface, we adopt the LJ parametrization from Heinz et al. [41] (segmented lines), which was specifically optimized for classical simulations of the metal–water interface, and the Universal force field (UFF) from Rappe et al. [42] (continuous line).

Apparently, the UFF parametrization for the gold surface yields relatively low variability in the density profiles for the three water models. When we adopt the Heinz parametrization, the profiles change significantly, which reflects a considerably stronger metal–electrolyte interaction, with the LJ well depth parameter $\varepsilon_{\mathrm{Au}}$ = 5.29kcal/mol compared to $\varepsilon_{\mathrm{Au}}$ = 0.039kcal/mol in UFF. We recall that under the default (Lorenz–Berthelot (LB)) mixing rules, the cross terms are generated as the geometric mean $\varepsilon_{ij}=\sqrt{\varepsilon_{i}\varepsilon_{j}}$, therefore, changing the gold self‐parameters directly propagates to all Au–water and Au–ion interactions.

Additionally, we observe that the cation peak is located between 2 and 3Å from the metal surface, that is, within the same region as the oxygen peak of the first water layer. This indicates that the cations are found directly at the Inner Helmholtz plane (IHP). The cation accumulation becomes more pronounced at negative electrode charge and we attribute this as the cause of the high differential capacitance, as discussed in Section 2.2.

This tendency for 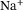 to accumulate directly at the surface is not specific to DRISM but is also observed in explicit molecular dynamics simulations. In a recent experimental and theoretical work from Alfarano et al. [34], it was found that 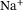 and 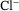 behave asymmetrically at the Au–water interface. Under negative bias, 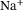 enters the IHP already at very small potentials and partially loses one hydration water, not because of strong 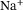
–Au bonding, but because the applied negative field disrupts the interfacial 2D hydrogen‐bond network, making insertion energetically favorable. In contrast, under positive bias, 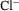 remains fully hydrated in the Outer Helmholtz plane (OHP) over a broad potential range and only desolvates at high positive potentials.

Given the approximations on which DRISM is based, we cannot expect it to capture the exact interfacial 2D hydrogen‐bond network and its disruption under applied potential. However, we still consider the ionic density profiles obtained from explicit MDs as a useful benchmark for the model. In Figure 4, we compare the density profiles obtained from a classical MD simulation of the Au(111) interface in contact with 1M NaCl at the PZC by Ntim and Sulpizi [43] with the corresponding DRISM results.

**FIGURE 4:**
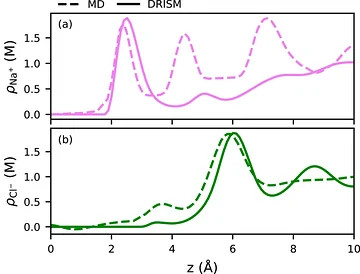
Density profiles for the Au(111) facet in contact with 1M NaCl in water. The profiles represented as dotted lines are taken from a classical MD simulation of Ntim and Sulpizi [43] using a polarizable force field for Au. The continuous profiles are obtained from DRISM using the mSPCE water model, the Heinz parametrization for the Au slab, and the Joung and Cheatham parametrization for the ions. In both cases, the electrode is at the PZC. Although the parametrization of the pair potentials is not identical, there is qualitative agreement in the positions of the first peaks for sodium and chloride.

As shown in panel (a), the position of the first 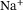 peak agrees well between the DRISM and MD results. Cation accumulation at the IHP has also been observed by Serva et al. [44] for the Au(100) facet, where the 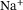 peak height was found to increase with the metallic character of the electrode. Interestingly, for the anion (panel (b)), we observe the main density peak at ≈6Å, in correspondence with the second water layer, and a minor peak at ≈3.5Å. The positions of both peaks are in qualitative agreement with the MD simulation. Also in this case, there is an agreement with a similar profile by Serva et al. [44].

From the classical theory of the electrical double‐layer [45], however, small cations such as 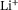 and 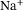 are known to typically retain their solvation shell and reside at the OHP, rather than populating the IHP, directly at the surface.

One potential limitation of the QM/RISM implementation in Quantum Espresso is the use of LB mixing rules to construct the cross pair‐potential parameters [23]. In the GolP‐CHARMM force field [46], which is specifically parameterized for the Au–water interface, the mixing rules are applied only to generic species, whereas the metal–water interactions are treated with pair‐specific parameters fitted to reproduce DFT adsorption energies of a single water molecule. In the classical MD studies of Hughes and Walsh [35] and Park and McDaniel [37], both employing GolP‐CHARMM, no cation accumulation directly at the interface is observed. In another recent work from Shin et al. [3], for an Ag(111) electrode, 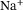 does not exhibit specific adsorption at cathodic potentials but remains solvated in the OHP, preserving its first hydration shell (coordination number ≈ 6) and avoiding direct contact with the Ag(111) surface. In this case, the metal–electrolyte interaction was modeled using first‐principles‐derived Buckingham‐type van der Waals potentials for Ag–water and Ag–ion interactions. We also note that an extensive study by Berg et al. [47] concluded that the LJ pair potential itself is insufficient for the Au–water interaction, and that alternative potentials such as the Morse or Buckingham potentials are more suitable options. Considering the different observations and conclusions of the studies discussed above, the microscopic structure of the electrical double layer at metal–aqueous interfaces seems still not fully understood, and several details remain open for debate.

It is therefore not straightforward to establish a unique benchmark for the DRISM results. However, a deeper investigation is important because an accurate description of the ionic density profile has a significant impact on the predicted physical properties of the double layer. As shown in Section 2.2, within the QM/DRISM method, the strong accumulation of 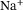 cations at the IHP leads to a large capacitance spike at negative electrode potentials, which is typically not observed in experimental cyclic voltammograms (CV) [48] and electrochemical impedance spectroscopy [49, 50]. For this reason, we proceed by examining the EDL structure predicted by DRISM, when pair‐specific parameters are used, instead of the default LB mixing rules.

In Figure 5a, we compare the density profiles at the PZC and for a small electrode surface charge obtained at a lower electrolyte concentration of 0.01M with the Au Heinz / mSPCE NaCl Joung parametrization. For the segmented curves, the default LB mixing rules are used and the sodium ion exhibits a defined peak within the IHP. This peak is also present for a positive surface charge applied of $\sigma =+0.27\;\mu C/{\text{cm}}^{2}$, in panel (c); at positive electrode charge, we expect the electrostatic repulsion to dominate and we therefore consider this peak as unphysical. By selectively decreasing the LJ interaction (see Figure S2) between gold and sodium (continuous line), we observe, as expected, the disappearance of the first sodium peak.

**FIGURE 5:**
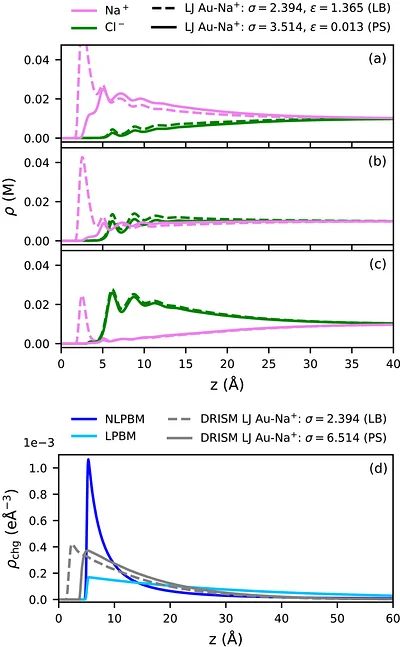
Density profiles for Au(111) facet in contact with 0.01M electrolyte. The calculations are performed at a surface charge of $\sigma =-0.27\;\mu C/{\text{cm}}^{2}$, PZC, $\sigma =+0.27\;\mu C/{\text{cm}}^{2}$, and $\sigma =-8.33\;\mu C/{\text{cm}}^{2}$ in (a), (b), (c), and (d), respectively. The segmented lines are computed with the Au Heinz/mSPCE/NaCl Joung parameterization and the default LB mixing rules. For the continuous lines, pair‐specific parameters are given for the Au–Na LJ interaction. In (d), results from the nonlinear and linear PB model are also shown for comparison.

These results suggest that DRISM is, in principle, capable of predicting the exclusion layer at the interface. As discussed before, the formation of such a layer arises from a complex interplay between the surface electronic structure, specific ion–surface interactions, and the rearrangement of the hydrogen‐bond network in the interfacial solvent. Within the QM/DRISM framework, these effects are incorporated only at the level of site–site correlation functions and approximate closure relations. Therefore, the emergence of an exclusion layer may reflect an effective description rather than a fully faithful representation of the underlying molecular mechanisms. Nevertheless, from a modeling perspective, the parameters could be empirically tuned to reproduce desired interfacial properties, such as ion depletion or trends in capacitance.

Within the PB family of models, the exclusion layer is typically introduced through an empirical ionic cavity correction (also referred to as a Stern‐layer correction) [11, 12, 32, 51]. A similar strategy can be used in DRISM by increasing the effective LJ repulsion radius between the electrode and the ions, which shifts ions away from the surface and generates an exclusion layer. Figure 5d compares this behavior with the linear and nonlinear PB models. Increasing the Au–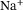 LJ repulsion radius indeed produces an exclusion layer analogous to the Stern‐layer correction in PB.

Although PB cannot reproduce the molecular‐scale structure of the compact layer, it is expected to describe the diffuse region more reliably in the dilute limit. In this regime, the electrolyte approaches an ideal gas of point ions in a dielectric continuum, and the ionic density decays exponentially away from the surface. As we can see DRISM reproduce the expected exponential decay at large distances from the surface, with a charge density which is intermediate between the linear and nonlinear PB models.

### 2.2 Differential Capacitance

The differential capacitance quantifies how the surface charge responds to changes in the applied potential. It has been extensively studied and remains one of the primary experimental probes of the structure of the electrical double layer.

In Figure 6, we report the differential capacitance as a function of the electrode potential relative to the PZC for the Au(111) surface in NaCl and HCl($H_{3}O^{+}{\mathrm{Cl}}^{-}$) electrolytes at 1M, using different parameters. For NaCl, we find that the differential capacitance increases steeply at negative electrode potentials.

**FIGURE 6:**
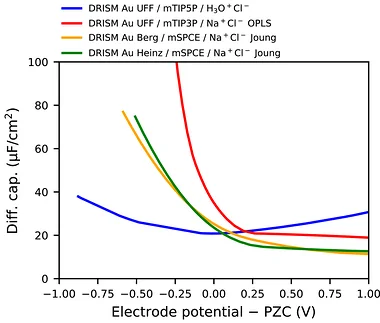
Differential capacitance for the Au(111) facet in contact with 1M aqueous electrolyte. For $H_{3}O^{+}$ (blue curve), the differential capacitance behaves as previously reported [30]. When 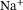 is used as a cation, the strong peak discussed in the previous section causes a divergence in the computed differential capacitance at negative electrode potentials.

Experimentally, this steep increase in the differential capacitance is observed at positive electrode potentials and is ascribed to specifically adsorbed anions [48, 52]; however, the measurements are typically done between 0.01 and 0.1M concentration. In the study of Devanathan and Ramakrishnaiah [49], differential capacitance was measured at 1M concentrations for different metals and different electrolytes; capacitance spikes at negative electrode potentials were also not observed. Furthermore, for a non‐adsorbing electrolyte, the differential capacitance is expected to exhibit a hump near the PZC at high electrolyte concentrations [51].

We therefore regard this excessively large differential as unphysical, and we trace it back to the strong peak in the sodium density profile discussed in the previous Section 2.1.2. Notably, the Au UFF / mTIP3P / NaCl Jensen parametrization, which corresponds to the weakest Au–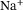 LJ interaction (ε=0.0044kcal/mol), yields the largest capacitance. This result highlights the important role of the Au–$H_{2}O$ interaction in preventing excessive accumulation of 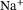 at the interface. In the Au UFF / mTIP3P parametrization the Au–$H_{2}O$ interaction is relatively weak, whereas it is stronger in the Au Heinz / mSPCE parametrization. We also tested the pair‐specific Au–$H_{2}O$ LJ parametrization optimized by Berg et al. [47]. Although this model employs Au–O LJ parameters similar to those of the Heinz parametrization, it introduces a predominantly repulsive Au–H interaction. Nevertheless, the resulting differential capacitance is largely unchanged. The Heinz and Berg parametrizations lead, however, to different solvation contributions in the adsorption energy of CO, as discussed in Section 2.3.

We mention that the increase of the capacitance at negative potentials was observed in a previous study employing the QM/ESM‐RISM framework, by Karnes et al. [53] for the Cu(111) and Cu(100) facets in contact with the mTIP5P water and different electrolyte ion species, where they show a clear asymmetry in the charging curves with respect to the PZC (see their Supporting Information, and Figure S4, where we replot the charging curve and the respective differential capacitance). In Figure S3, we additionally show the differential capacitance obtained using the LJ parameters of Haruyama et al. [25] for Pt, which were fitted to reproduce the interaction energy between a Pt surface and a Xe atom from a DFT calculation. Even with these parameters, we still observe pronounced cation accumulation at the interface, leading to divergent differential capacitance values at negative electrode potentials.

In contrast, when $H_{3}O^{+}$ is used as the cation in the DRISM electrolyte, this behavior is not observed. In DRISM, 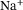 is represented as a single‐site classical particle with its positive charge located at the ion center, which allows it to approach the electrode surface closely. $H_{3}O^{+}$, instead, is modeled as a multi‐site species with the positive charge distributed over the three hydrogen atoms. As a result, the effective charge cannot approach the interface as closely, leading to a smaller accumulation of positive charge at the electrode surface. In agreement with Demeyere and Skylaris [30], we also observe fluctuations in the computed Fermi level, particularly for simulations using the mTIP5P water model and the $H_{3}O^{+}$ cation and we found the convergence significantly more difficult, requiring and a convergence threshold for the 3D‐RISM solver of $10^{-6}\;\text{Ry}$ instead of $10^{-7}\;\text{Ry}$, as reported in the methods Section 4. Moreover, we report that when using the mTIP5P model and the $H_{3}O^{+}$ cation with DRISM, some calculations did not converge. This behavior may be linked to the larger number of interaction sites in both mTIP5P water and $H_{3}O^{+}$, which increases the complexity of the site–site correlations.

In Figure 7a, we examine the dependence of the differential capacitance on the NaCl electrolyte concentration. As the concentration decreases, the differential capacitance systematically decreases as well. Notably, the divergence observed at negative electrode potentials is strongly suppressed at lower concentrations and, in particular, it becomes negligible at 0.1M and is no longer present at 0.05M.

**FIGURE 7:**
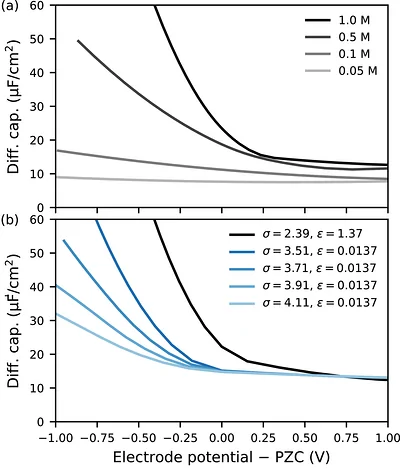
Differential capacitance for the Au(111) facet in contact with aqueous NaCl electrolyte: (a) at different electrolyte concentrations; (b) with different pair‐specific parameters for the Au–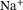 interaction. The black curve corresponds to the default LB mixing rules, while for the other curves, the Au–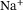 parameters are changed as in the legend. The remaining parameters are obtained using the default mixing rules for the Au Heinz/mSPCE/NaCl Joung parametrization.

As we discussed in the previous Section 2.1.2, the pair‐specific metal–cation parametrization plays an important role in determining the density profiles. In Figure 7b, we therefore analyze the dependence of the differential capacitance on the pair‐specific Au–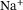 LJ parameters, again using the Au Heinz/mSPCE/NaCl Joung parametrization as a reference.

The black curve corresponds to the default LB mixing rules. For the other curves, we selectively reduce the ε parameter by two orders of magnitude and progressively increase σ by 0.2Å for the Au–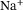 pair. Upon weakening the attractive interaction (smaller ε), the differential capacitance at the PZC decreases markedly. A further increase of the repulsive radius σ leads to a progressive reduction of the capacitance at negative potentials, whereas the positive branch remains largely unaffected. This highlights the crucial role of the Au–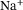 interaction in determining the differential capacitance, and the limitation of the LB mixing rules.

We next examine the influence of the cation species on the differential capacitance. In Figure 8a, we compare the differential capacitance at the PZC predicted by DRISM for different electrodes in contact with various electrolytes at a concentration of 0.05M, with the experimental measurements reported by Garlyyev et al. [54] and Xue et al. [55]. Experimentally, the differential capacitance at the PZC increases along the series 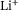, 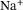, $K^{+}$, ${\mathrm{Rb}}^{+}$, and ${\mathrm{Cs}}^{+}$, a trend attributed to the corresponding increase in cation hydration energy.

**FIGURE 8:**
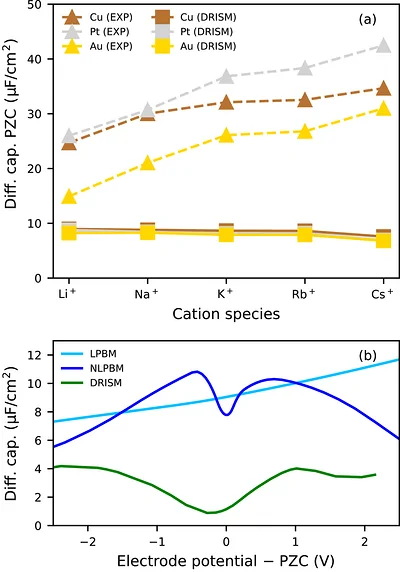
(a) Differential capacitance at the PZC for the (111) facet of Cu, Pt, and Au for different cationic species at 0.05M concentration (adopting the Heinz/mSPCE/ion Joung parametrization) The experimental data (triangle markers) [54, 55] show an increasing trend with increasing cationic species. The computed differential capacitance from DRISM (square markers), in contrast, is mostly constant, between 8 and $9\;\mu F/{\text{cm}}^{2}$. (b) Differential capacitance for the Au(111) facet in contact with 0.01M aqueous electrolyte. In green, the differential capacitance from DRISM for Au Heinz/mSPCE/KBr Joung parameters. KBr leads to better convergence of the Fermi level (probably due to its larger ionic radius), so we present it instead of NaCl. The differential capacitance from the linear (light blue) and nonlinear (blue) PB models is also shown for comparison.

We note, however, that the experimental values for strongly adsorbing metals such as Pt should be interpreted with caution, as different measurement techniques can yield considerably different capacitances at the PZC as initially highlighted by Zhang and Huang [56], and further discussed by Fröhlich and Koper [57].

In contrast to experiment, the DRISM results do not show a pronounced cation‐dependent trend with the chosen parameterization: the predicted differential capacitances lie within a narrow range of 8–$9\;\mu F/{\text{cm}}^{2}$ for all cation species considered. For completeness, we also highlight, however, that a recent experimental study by Adnan et al. [48] on Au(111) similarly reports no significant dependence of the differential capacitance at the PZC on the identity of the cation, suggesting that further experimental clarification is required before a final conclusion can be drawn.

To further assess the ability of DRISM to capture the nonlinear behavior of the differential capacitance near the PZC, we also examined a lower electrolyte concentration of 0.01M. At this concentration, the simulation requires a significantly larger cell, which increases the fluctuations in the computed Fermi level; consequently, stronger smoothing is necessary. Therefore, the resulting shape of the differential capacitance should be interpreted in a qualitative manner (see the Supporting Information). In Figure 8b, we show the resulting comparison with the PB model.

Despite the ionic density profile at low electrolyte concentration being in qualitative agreement with the PB model (Figure 5), DRISM predicts a lower capacitance than both linear and nonlinear PB. This suggests that, at low concentration, DRISM does not fully capture the diffuse‐layer contribution to the capacitance. However, a reduced diffuse‐layer capacitance alone is not sufficient to explain the overall underestimation. A possible explanation may lie in the Helmholtz‐layer contribution, in particular in the response of the interfacial water layer. The shape of the differential capacitance of Au(111) in contact with dilute aqueous NaCl has recently been investigated by Zhen et al. [58]. In particular, they showed that the pronounced experimentally observed double‐peak structure originates primarily from the orientational polarization of the adsorbed water layer. The absence of this peak structure in the DRISM capacitance therefore suggest that the model—within the tested parametrizations—does not fully capture the Helmholtz capacitance associated with the interfacial water layer. The characteristic nonlinear behavior around the PZC seems nevertheless to be captured at a qualitative level. We also observe that the minimum in the differential capacitance is slightly shifted toward negative potentials rather than aligning with the PZC. However, this deviation may arise from the fitting procedure rather than reflecting a physical interfacial effect (see the charging curve and the respective fit in Figure S5).

### 2.3 Adsorption Energies

Finally we analyze the impact of the DRISM parametrization on adsorption energies using CO as a prototypical probe, motivated by its central role in the electrocatalytic CO_2_ reduction reaction (eCO_2_RR).

In Figure 9, we present the adsorption energies at the PZC in solvent water only, for different parametrizations, and we compare them to the one computed with the Poisson model in VASPsol++. To highlight the effect of solvation, we take the adsorption energy in vacuum as an internal reference. For the Poisson model, we observe a minimal stabilization with respect to the vacuum reference, and no significant trend with respect to the adsorption site. These findings are in agreement with the previous study of Heenen et al. [13], where the solvation contribution to the CO adsorption energy was found to be small and relatively independent of the adsorption site. On the other hand, for DRISM, we observe a significant dependence on the parametrization, with a difference of up to ≈6kcal/mol. The parametrization from Heinz is found to destabilize the CO adsorption, whereas the UFF parametrization has a stabilizing effect. This can be explained by the Au–O LJ parametrization; for the Heinz/mSPCE parametrization, the metal–oxygen interaction energy is $\varepsilon_{\text{Au--O}}\approx 0.9\;\text{kcal}/\text{mol}$, while for the UFF/mTIP3P parametrization it is much lower $\varepsilon_{\text{Au--O}}\approx 0.08\;\text{kcal}/\text{mol}$. The CO adsorption is disrupting the structure of the double layer at the interface, and the stronger Au–O interaction in the Heinz parametrization leads to a larger solvation penalty for the adsorption. This findings are consistent with the previous studies [29, 30]. A weaker adsorption energy for CO in electrolyte was also observed experimentally [59] and ascribed to the competition between CO and water for the adsorption sites. We also tested the parametrization from Berg et al. [47], which is similar to the Heinz parametrization for the Au–O interaction, but introduces a predominantly repulsive Au–H interaction, which decrease the solvation energy of ≈1kcal/mol. We further notice that the difference between the three DRISM parametrizations is mostly constant and shows an analogous trend of increasing stabilization in the order: on top, bridge, and fcc/hollow.

**FIGURE 9:**
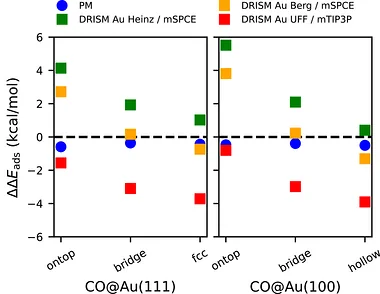
Solvation contribution to the adsorption energy of CO at the Au(111) and Au(100) facets, at the PZC, for different DRISM parametrizations and for the Poisson model in VASPsol++.

To test the effect of the mobile electrolyte ions, we computed the adsorption energies in 1M NaCl electrolyte, for two different surface sites CO@Au(100) ontop and hollow. In Figure 10a, we show the dependence of the adsorption energy on the surface charge density.

**FIGURE 10:**
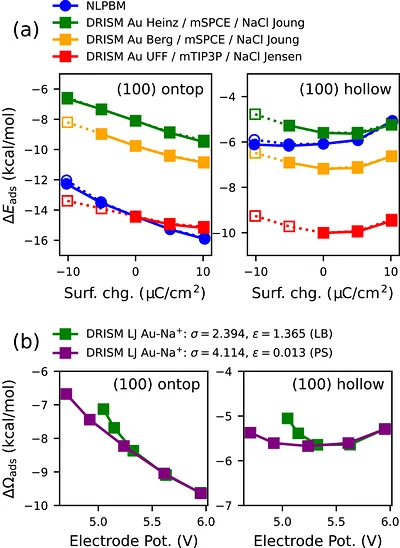
(a) Adsorption energy of CO at the Au(100) facet in contact with 1M electrolyte, as a function of the surface charge density. For the hollow markers, the adsorption energy is computed from single point calculations, with structures optimized at the PZC, since for some datapoints the full convergence of the atomic forces was not achieved, as discussed in the main text. (b) Grand canonical adsorption energy of CO at the Au(100) facet in contact with 1M NaCl electrolyte, as a function of the electrode potential (relative to the electrolyte bulk), using the default LB mixing rules (green), and for a reduced Au–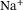 interaction (purple).

We highlight that for some datapoints, it was not possible to achieve full convergence of the atomic forces under the defined threshold value. We ascribe this behavior to the strong accumulation of 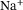 close to the interface at negative surface charge densities, which leads to a very steep energy landscape and makes the optimization process more difficult. Therefore, we also report the adsorption energy computed from single‐point calculations, with structures optimized at the PZC, with hollow markers and dotted lines.

Given the small differences between the two approaches, we regard the overall trends as reliable, even for the points where full convergence was not achieved. The different parametrizations show a very similar behavior, with analogous solvation trends as observed in Figure 9. Similar trends are also observed for the PB model.

A different picture emerges when we examine the dependence of the adsorption energy on the electrode potential, as shown in Figure 10b. In green, we show the Heinz/mSPCE/NaCl Joung parametrization, with the conventional LB mixing rules, while in purple we show the same parametrization but with a reduced Au–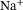 interaction, as in Figure 7. The asymmetry encountered for the Heinz/mSPCE/NaCl Joung parametrization, in the capacitance curve at negative potentials is now reflected in the adsorption energy. When decreasing the Au–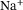 interaction, the adsorption energy becomes more symmetric with respect to the PZC, and we regard it as more accurate, since, as discussed in Section 2.2, the steep increase of the differential capacitance at negative potentials is not observed experimentally.

## 3 Conclusion

In this work, we assessed the dielectrically consistent DRISM embedding for modeling the EDL and compared its predictions with explicit MD data and continuum PB models. For the gold–water interface, DRISM reproduces the main features of the solvent density profiles and yields a qualitatively reasonable microscopic description of the interfacial liquid structure. For ionic density profiles, the method also captures the position of the main peaks observed in explicit MD simulations. Additionally, our results show that the predicted structure of the compact layer depends strongly on the parametrization of the metal–ion interactions. With the default LB mixing rules, 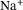 can accumulate too strongly in the IHP, which leads to divergent differential capacitance at negative electrode potentials for concentrated NaCl electrolytes, a behavior that we regard as unphysical. We show that by introducing pair‐specific Au–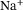 parameters, this excessive cation accumulation is reduced, and this is reflected in a more symmetric differential capacitance curve.

We expect different water models to have an impact, especially on the Helmholtz layer capacitance. The mTIP5P water model has two more lone‐pair sites compared to mSPCE and mTIP3P, which offer, in principle, greater tunability. However, we observed convergence problems for these calculations, which limited the accuracy of the differential capacitance curves we obtained to a degree that did not allow us to comprehensively assess the different behavior of this water model.

In the dilute limit, DRISM recovers the expected exponential decay of the diffuse charge distribution, and charge profiles lie between the linear and nonlinear PB model.

For CO adsorption, the solvation contribution depends strongly on the chosen metal–water parameters, with differences up to 6kcal/mol but the trends across different adsorption sites are consistent across different parametrizations. The dependence of the adsorption energy on the electrolyte is more evident when examining the adsorption energy as a function of the electrode potential, where the asymmetry in the adsorption energy follows the same behavior observed in the differential capacitance. Also, in this case, we suggest that the introduction of pair‐specific parameters for the Au–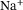 interaction leads to a more symmetric behavior of the adsorption energy with respect to the PZC, which we regard as more accurate.

Our analysis highlights some aspects that require particular care when simulating the electrode–electrolyte interface with QM/DRISM. The physical behavior of a given parametrization should be assessed directly from the solvent and ionic density profiles, and, whenever possible, compared with explicit MD or AIMD reference data. Comparison with experimentally accessible observables, such as the differential capacitance; however, offer a more unbiased and hence robust means of validation. This comparison should be considered for each specific electrode–electrolyte system under investigation, since the relevant interfacial mechanisms can depend strongly on the metal, ion species, and electrolyte concentration. Finally, QM/DRISM offers a far more advanced atomistic picture than the more conventional PB models, however, as in QM/MM embedding, the electrolyte is described through classical particle distributions; therefore processes requiring explicit electronic structure in the electrolyte, such as charge transfer or bond formation between electrode and electrolyte species, cannot be described explicitly.

Overall, our results demonstrate that the adoption of pair‐specific parameters within the QM/DRISM framework can improve the flexibility of the model. We believe that a systematic optimization of the pair‐wise parameters, could significantly enhance the accuracy of the model and can be objective of future work.

## 4 Methods

We used the Vienna ab initio Simulation Package [60] (VASP) with the VASPsol++[32] Poisson–Boltzmann (PB) model and Quantum ESPRESSO [61, 62] with the built‐in implementation of the reference interaction‐site model (RISM) [23, 31]. For the calculations with VASP, we built‐in PAW pseudopotentials, while with Quantum ESPRESSO, we used the PAW pseudopotentials from the PSlibrary [63]. The initial atomic structures were generated using the Atomic Simulation Environment (ASE) [64].

### 4.1 Density Profiles and Differential Capacitance

For computing the density profiles and the differential capacitance, we chosen a 1×1×6 slab model, with the default experimental lattice parameters in ASE. To ensure that the cell size would not affect the results, we compared the density profiles obtained from a slab with a larger surface 3×3. As shown in Figure S1, the profiles overlap well at both high and low electrolyte concentrations, proving that a 1×1 surface cell is sufficient to investigate the EDL's properties within the QM/ESM‐RISM framework. We employed the PBE density functional [65] and Gaussian smearing with a broadening parameter of 0.2eV (≡0.0147Ry). We employed a plane wave cutoff of 500eV (≡36Ry, with an additional ×8 cutoff for the electron density in Quantum ESPRESSO). For sampling the Brillouin zone, we used a 11×11×1 Γ‐centered k‐point grid. For the VASP calculations, we used an energy threshold of $10^{-6}\;\text{eV}$ for the electronic energy, while for Quantum ESPRESSO we set the threshold to $10^{-8}\;\text{Ry}$. For RISM, two additional thresholds must be defined: the first establishes the convergence of the 1D‐RISM calculation, for which we used a value of $10^{-8}\;\text{Ry}$; the second is related to the 3D‐RISM calculation, which we set to $10^{-7}\;\text{Ry}$. We used a tighter convergence than previously reported [25], since we found the Fermi level to be very sensitive to the 3D‐RISM convergence. We report, however, that for the mTIP5P water model, it was not possible to reach the desired convergence, and therefore, we increased the threshold to $10^{-6}\;\text{Ry}$. Finally, for RISM, an additional plane wave cutoff is required to expand the 3D particle densities; we used 300Ry.

In both the implicit electrolyte models, the electrode potential is defined with respect to the electric potential in the electrolyte bulk, according to the following expression:

(1) $$\Phi_{\text{el.de}}=\phi_{\text{el.yte}}-\frac{{\bar{\mu }}_{e}}{q_{e}}$$

where ${\bar{\mu }}_{e}$ is the electrochemical potential of the electrons in the metal electrode, that is, the Fermi level, $q_{e}$ is the elementary charge, and ϕ is the electric potential in the electrolyte bulk region.

The differential capacitance is computed by performing several calculations at different surface charges, that is, by changing the number of electrons in the system. The surface charge is defined as the excess of electronic charge in the system divided by the surface area of the cell. For the PB model implementation in VASP, since the electrolyte is present on both sides of the cell, the surface area must be multiplied by two. The ESM‐RISM implementation in Quantum ESPRESSO allows for a non‐periodic vacuum/slab/electrolyte setup; therefore, the surface area is just the one in contact with the electrolyte. The resulting charging curve is fitted with a cubic spline and differentiated to obtain the differential capacitance. A different level of smoothing might be required, given that even small fluctuations in the computed Fermi level lead to large discontinuities in the first derivative (as previously reported [30]).

### 4.2 Adsorption Energies

For the adsorption energy, we used the RPBE density functional [66] along with the D3 dispersion correction (with “zero damping” function) [67] following previous studies [13, 68] on the gold electrode interface. We used the plane wave cutoff of 600eV (≡44Ry, with an additional ×8 cutoff for the electron density in Quantum ESPRESSO). To construct the slab we employed an optimized lattice constant for a 1×1×1 cubic cell (with a 21×21×21 k‐point mesh). The resulting lattice constants are 4.172Å for VASP, and 4.196Å for Quantum ESPRESSO. The small difference can be ascribed to the different pseudopotentials. The slab model consists of a 3×3 surface cell with four layers, where the two bottom layers are fixed to the bulk positions and the two top layers are allowed to relax. The Brillouin zone is sampled with a 3×3×1 Γ‐centered k‐point grid, and Gaussian smearing with a broadening parameter of 0.1eV (≡0.0073Ry) is used. The force convergence threshold for the geometric optimization is set to 0.01eV/Å for VASP and 0.002Ry/Bohr for Quantum ESPRESSO. As we highlight on the main text, for some of the Quantum ESPRESSO calculations, denoted with the hollow markers in Figure 10a, was not possible to achieve the desired convergence of the forces, and therefore, we report the adsorption energy computed from single point calculations, with the geometry optimized at the PZC. The adsorption energy is computed as

(2) $$\Delta E_{\text{ads}}=E_{\text{CO}@\text{slab}}-E_{\text{slab}}-E_{\text{CO}}.$$

The solvation contribution to the adsorption energy is then computed as the difference between the adsorption energy in solvent and the adsorption energy in vacuum

(3) $$\Delta \Delta E_{\text{ads}}=\Delta E_{\text{ads,solvent}}-\Delta E_{\text{ads,vacuum}}.$$

The for the grand‐canonical adsorption energy, we also include the contribution from the change in the number of electrons in the system, as follows:

(4) $$\Delta \Omega_{\text{ads}}=\Delta E_{\text{ads}}-\Delta N_{e}\cdot {\bar{\mu }}_{e}.$$

We point out, however, that for computational convenience, we did not include the vibrational contribution to the adsorption energy. Since our main focus is on the solvation contribution, we expect this approximation to be reasonable, and not affect the overall trends and discussion.

### 4.3 Electrolyte Models

The main parameters of the PB model are the dielectric constant of the solvent, in our case water, the radius of the dielectric cavity, the radius of the ionic cavity, and the maximum ionic packing diameter. We used the default settings, as suggested in the original implementation [32].

Within the RISM formalism, the pairwise potentials among the interaction sites must be provided. In the specific implementation by Nishihara and Otani [23], the pairwise potentials are given in the form of Lennard‐Jones 12–6 expression and the Lorentz–Berthelot combination rules are adopted $\sigma_{AB}=\frac{\sigma_{AA}+\sigma_{BB}}{2}$, $\varepsilon_{AB}=\sqrt{\varepsilon_{AA}\cdot \varepsilon_{BB}}$. The use of pair‐specific parameters for the LJ interaction between the explicit system and the implicit electrolyte is described case by case in the main text (to define pair‐specific parameters we used our own modified version of Quantum ESPRESSO, since at the present moment only the Lorentz–Berthelot mixing rules are implemented in the official release).

For water, we use the SPCE [69], the TIP3P [70], and the TIP5P [71] models, with the modifications adopted in [25]. The modifications consist of additional LJ potentials for hydrogen atoms and lone charge pairs in the TIP5P model. The repulsive part of the LJ potential is needed to compute site–site particle correlations, which would otherwise diverge at short distances. To highlight this difference from the original models, we denote them as “modified”: mSPCE, mTIP3P, and mTIP5P. For the ions, we used the parameters optimized for the SPCE water model from the work of Joung and Cheatham [38], and the OPLS parametrization from Jensen and Jorgensen [39] in combination with the TIP3P and TIP5P water models. For the explicit QM system, we used the LJ parameters from the UFF [42] and from Heinz et al. [41].

Finally, for the dielectric correction in DRISM, we used the same settings as in the original implementation [31].

## Conflicts of Interest

The authors declare no conflicts of interest.

## Supporting information


**Supporting File 1**: chem71502‐sup‐0001‐SuppMat.pdf

## Data Availability

The data that supports the findings of this study are available in the supplementary material of this article.
