# Supplementary material for: Investigating the Electrochemical Double Layer with Quantum‐Chemical Simulations and Implicit Solvation Models
Source: Chemistry. 2026 Aug 11;32(41):e71502. doi: 10.1002/chem.71502 (PMC13729958; doi:10.1002/chem.71502)
Supplement: Supplementary file 1 [file CHEM-32-e71502-s001.pdf]

Supplementary Information: Investigating the Electrochemical Double Layer with Quantum-Chemical Simulations and Implicit Solvation Models

Alessandro Mangiameli[1] and Christopher J. Stein *[1,2]

[1]Department of Chemistry and Catalysis Research Center, TUM School of Natural Sciences, Technical University Munich, 85748 Garching, Germany
[2]Atomistic Modeling Center, Munich Data Science Institute, 85748 Garching, Germany

*christopher.stein@tum.de

1 Ionic Density Profiles for Different Cell Sizes

In figure 1, we report the density profiles for NaCl electrolyte in mTIP3P water for two different electrode cell sizes. The two colored lines correspond to a 1×1 Au slab, while the dotted lines correspond to a 3×3 Au slab. As we can see, the profiles overlap almost perfectly, justifying the use of a smaller cell to save computational time.

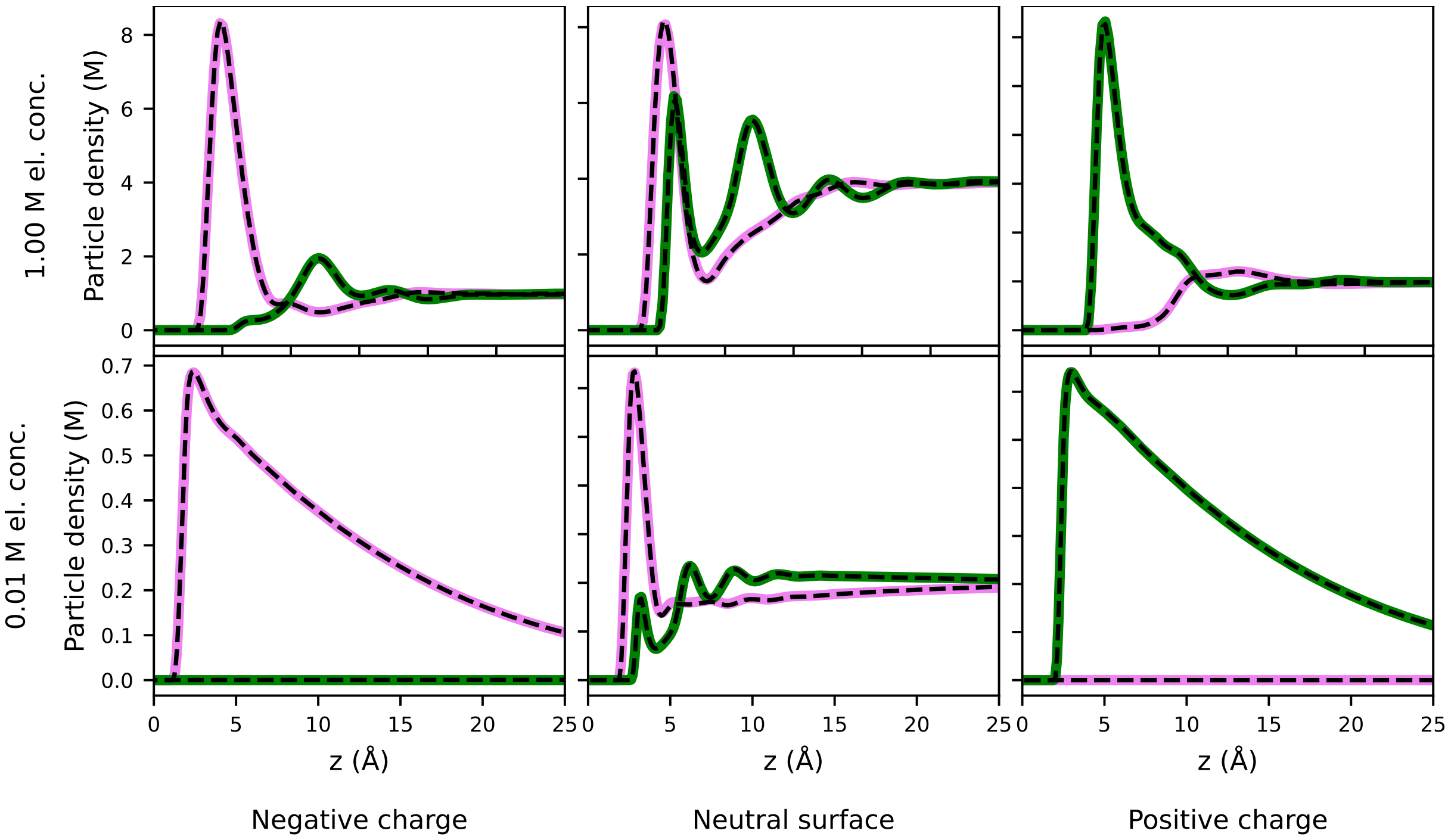

Figure 1: Comparison of the NaCl density profiles for a 1×1 Au slab continuous colored lines, and for a 3×3 Au slab, black segmented lines. The profiles overlap well at both high and low electrolyte concentrations, proving that a 1×1 surface cell is sufficient to investigate the EDL's properties.

2 Lennard-Jones Parameters

In Table 1 we report the Lennard-Jones parameters we adopted in our study in combination with the Lorentz–Berthelot mixing rules.

Implicit Sites	σ (Å)	ε (kcal/mol)
O / mSPCE	3.1660	0.1554
H / mSPCE	1.0000	0.0460
O / mTIP3P	3.1507	0.1521
H / mTIP3P	1.0000	0.0460
O / mTIP5P	3.1200	0.1600
H / mTIP5P	1.0000	0.0460
L / mTIP5P	1.8000	0.0460
Na^+ / Joung	2.1595	0.3526
Cl^- / Joung	4.8305	0.0129
Na^+ / OPLS	4.0700	0.0005
Cl^- / OPLS	4.0200	0.7100

Explicit Sites	σ (Å)	ε (kcal/mol)
Au / Heinz	2.6290	5.2900
Au / UFF	2.9337	0.0390
O	3.1200	0.1591
C	3.8300	0.0262

Table 1: Lennard-Jones parameters used in this study in combination with the Lorentz–Berthelot mixing rules.

3 Pair-specific Lennard-Jones Parameters

In figure 2 we report the Lennard-Jones potential for the Na-Au interaction. The black segmented curve corresponds to the default Lorentz–Berthelot mixing rules, while for the continuous colored curves the parameters are pair-specific. The figure highlights that two Lennard-Jones parameters σ and ε, adopted within the following functional form:

$$V_{\mathrm{LJ}}(r) = 4\varepsilon \left[\left(\frac{\sigma}{r} \right)^{12} - \left(\frac{\sigma}{r} \right)^{6} \right]$$

cannot be modified independently, as they are both involved in the position and depth of the potential well. As we can see, for the blue curve we decrease the ε parameter by a factor of 100, in order to reduce the strength of the Au-Na interaction, while the σ parameter is kept unchanged. This results in a significant shift of the repulsive wall of the potential. Therefore as shown by the red curve, in order to keep the position of the repulsive wall unchanged, we increase the σ parameter to 3.514 Å.

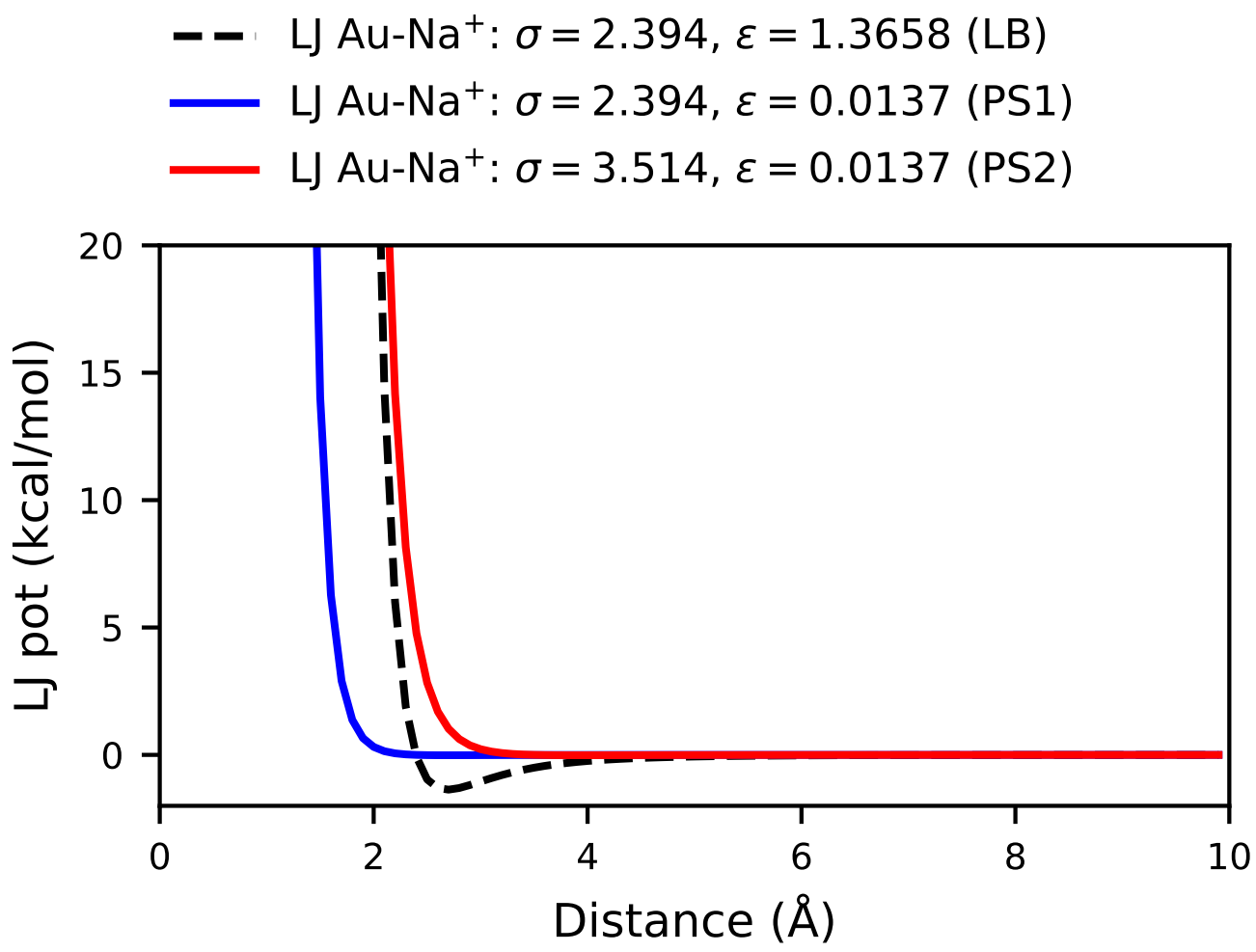

Figure 2: Lennard-Jones potential for the Na-Au interaction. The black segmented curve corresponds to the default Lorentz–Berthelot mixing rules, while the continuous colored curves correspond to pair-specific parameters. The blue curve shows a decrease in the ε parameter by a factor of 100, while keeping the σ parameter unchanged, resulting in a significant shift of the repulsive wall of the potential. The red curve shows the effect of increasing the σ parameter to 3.514 Å, in order to keep the position of the repulsive wall unchanged.

4 Differential Capacitance for other Systems

In figure 3 we present the differential capacitance for a Pt electrode in NaCl computed with RISM and DRISM, respectively. For the metal slab, we adopted the optimized Lennard-Jones parametrization from Haruyama [1] *et al.*

Similarly, in Fig 4 we present the charging curve and differential capacitance using the data taken from Karnes et al. [2].

These evidences further support the hypothesis that the origin o the high capacitance at negative potentials is due to the metal-cation parametrization.

5 Differential Capacitance Fitting at 0.01 M Concentration

In figure 5 we report the charging curve and the relative fitting for a Au(111) electrode in KBr electrolyte at 0.01 M concentration, computed with DRISM. The fitting is performed using smoothed cubic splines, and the resulting curve is used to compute the differential capacitance. As we can see, the noise in the Fermi level makes the fitting procedure more difficult, and the resulting curve is not as smooth as for higher concentrations, where the noise is less pronounced. We therefore interpret the shape of the differential capacitance at 0.01 M only at qualitative level.

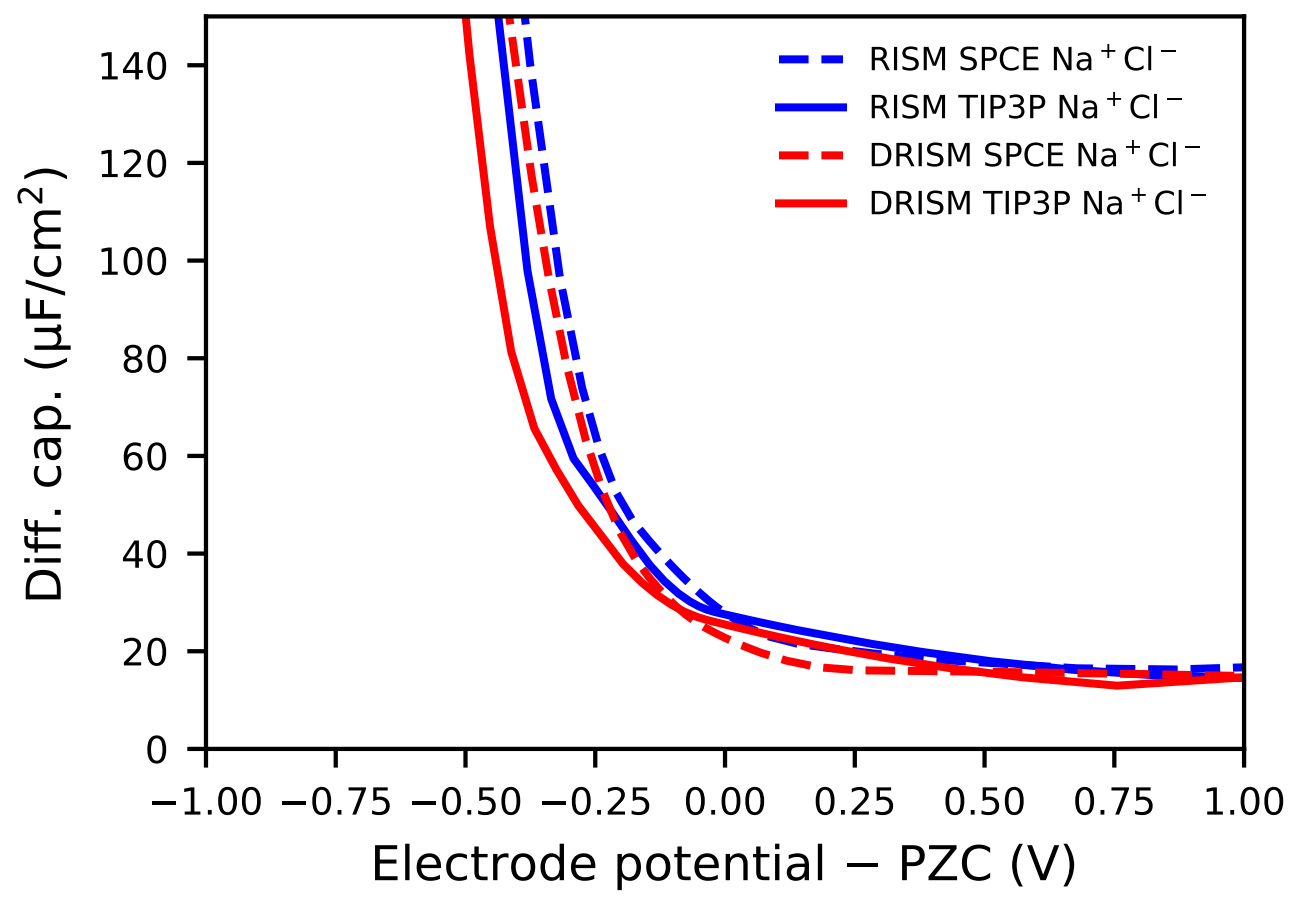

Figure 3: Differential capacitance for a Pt electrode in NaCl computed with RISM and DRISM, respectively. For the metal slab, we adopted the optimized Lennard-Jones parametrization from Haruyama et al. [1].

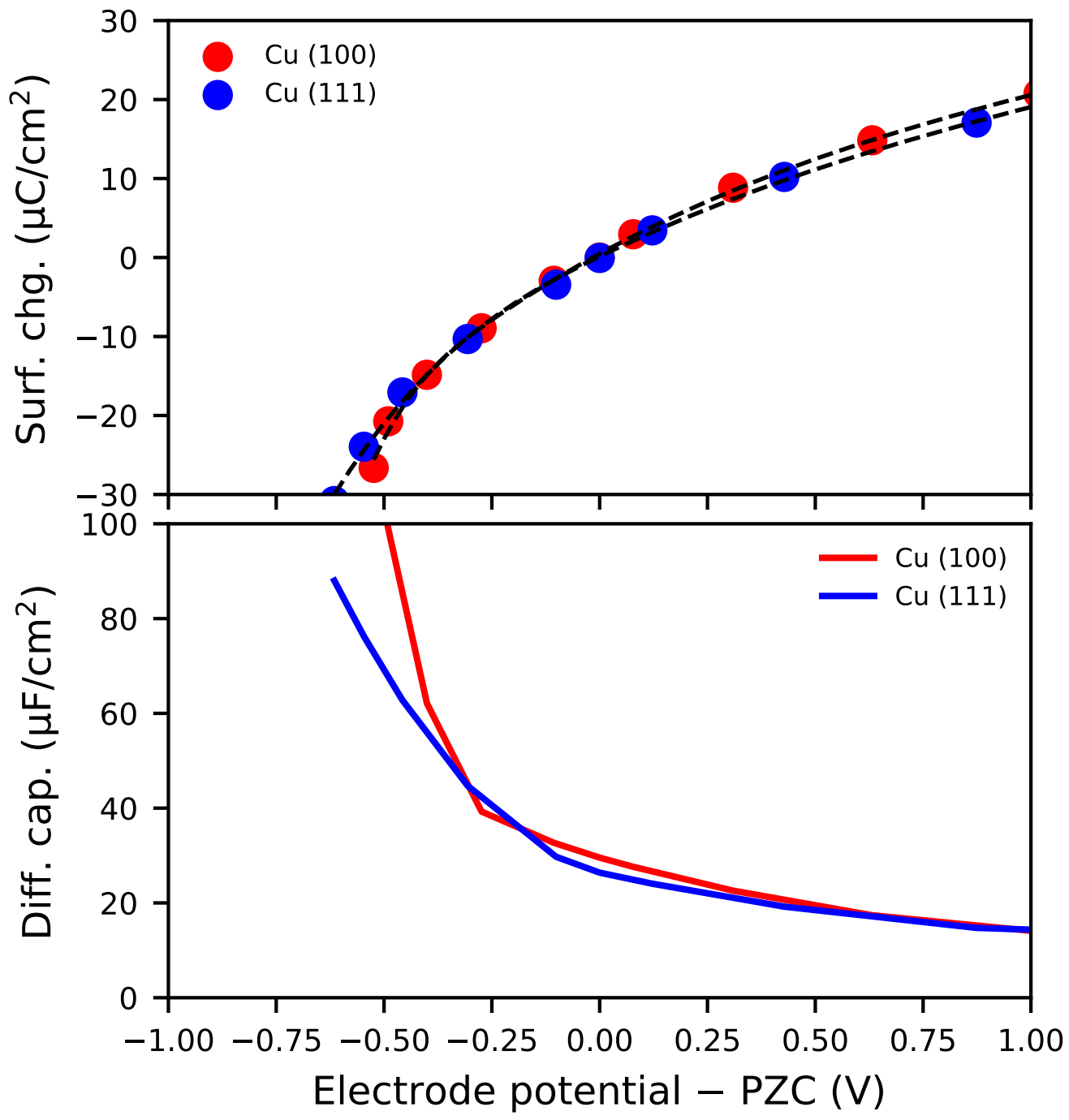

Figure 4: Charging curve and differential capacitance for a Cu electrode in KBr electrolyte, using the data taken from Karnes et al. [2].

6 Adsorption Energies in Vacuum

In Table 2 we report the CO@Au adsorption energies in vacuum for the different facets and sites. The values computed with the two software packages are in good agreement, with a maximum difference of $\approx 0.5\,\mathrm{kcal/mol}$ which can be attributed to the different pseudopotentials used in the two codes.

Site	VASP (kcal/mol)	QE (kcal/mol)
(111) ontop	−7.2	−7.0
(111) bridge	−5.8	−6.1
(111) fcc	−4.7	−5.1
(100) ontop	−13.9	−13.5
(100) bridge	−16.6	−16.6
(100) hollow	−5.6	−5.5

Table 2: Adsorption energies of CO on Au in vacuum for different facets and sites, computed with VASP and Quantum ESPRESSO.

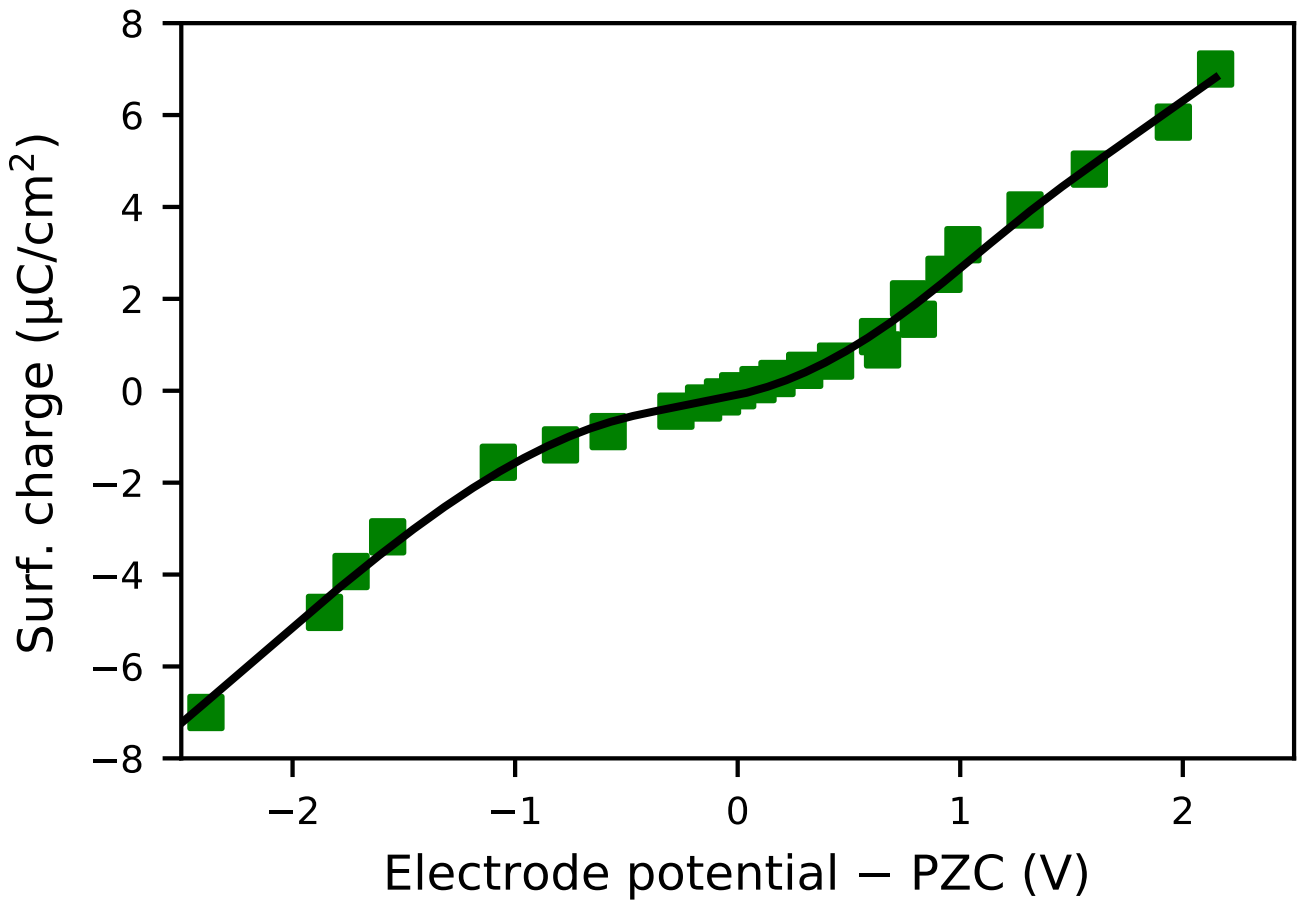

Figure 5: Charging curve and relative fitting for a Au(111) electrode in KBr electrolyte at 0.01 M concentration, computed with DRISM.

7 XYZ Structures

Bare Au slabs:
Au(111) (for profiles and capacitances)

```
6
Lattice="2.8849958217166707 0.0 0.0 1.4424979108583353 2.4984785065021797 0.0 0.0 0.0 61.7779474632004"
Properties=species:S:1:pos:R:3 pbc="T T T"
Au       1.44249791       0.83282646     -15.88897370
Au      -0.00000000       1.66565320     -13.53338442
Au       0.00000000       0.00000000     -11.17779514
Au       1.44249791       0.83282646      -8.82220586
Au      -0.00000000       1.66565320      -6.46661658
Au       0.00000000       0.00000000      -4.11102759
```

Au(111)

```
36
Lattice="8.900591307799893 0.0 0.0 4.450295653899946 7.708134587337402 0.0 0.0 0.0 47.267302187375954"
Properties=species:S:1:pos:R:3 pbc="T T T"
Au       0.00000000       0.00000000      -8.63365115
Au       2.96686374       0.00000000      -8.63365115
Au       5.93372749       0.00000000      -8.63365115
Au       1.48343187       2.56937937      -8.63365115
Au       4.45029562       2.56937937      -8.63365115
Au       7.41715936       2.56937937      -8.63365115
Au       2.96686374       5.13875874      -8.63365115
Au       5.93372749       5.13875874      -8.63365115
Au       8.90059123       5.13875874      -8.63365115
Au       1.48343187       0.85645979      -6.21121705
Au       4.45029562       0.85645979      -6.21121705
Au       7.41715936       0.85645979      -6.21121705
Au       2.96686374       3.42583916      -6.21121705
Au       5.93372749       3.42583916      -6.21121705
Au       8.90059123       3.42583916      -6.21121705
Au       4.45029562       5.99521853      -6.21121705
Au       7.41715936       5.99521853      -6.21121705
Au      10.38402310       5.99521853      -6.21121705
Au      -0.00008063       1.71313691      -3.83200267
Au       2.96684346       1.71308255      -3.83201426
Au       5.93387599       1.71312317      -3.83201443
Au       1.48333020       4.28218186      -3.83208321
Au       4.45041003       4.28227202      -3.83207643
Au       7.41719681       4.28215494      -3.83210085
Au       2.96695492       6.85168026      -3.83200079
Au       5.93368849       6.85153692      -3.83200152
Au       8.90059036       6.85154485      -3.83194172
Au      -0.00004993       0.00010534      -1.40323393
Au       2.96688960      -0.00003994      -1.40315533
Au       5.93376483       0.00006609      -1.40320228
Au       1.48325781       2.56949158      -1.40327753
Au       4.45034291       2.56941619      -1.40330058
Au       7.41727382       2.56941475      -1.40340817
Au       2.96683609       5.13870670      -1.40325636
Au       5.93380960       5.13870108      -1.40338529
Au       8.90055250       5.13860575      -1.40329373
```

Au(100)

```
36
Lattice="8.900591307799893 0.0 0.0 0.0 8.900591307799893 0.0 0.0 0.0 46.29366650421573"
Properties=species:S:1:pos:R:3 pbc="T T T"
Au       1.48343187       1.48343187      -8.14683421
Au       4.45029562       1.48343187      -8.14683421
Au       7.41715936       1.48343187      -8.14683421
Au       1.48343187       4.45029562      -8.14683421
Au       4.45029562       4.45029562      -8.14683421
Au       7.41715936       4.45029562      -8.14683421
Au       1.48343187       7.41715936      -8.14683421
Au       4.45029562       7.41715936      -8.14683421
Au       7.41715936       7.41715936      -8.14683421
Au       0.00000000       0.00000000      -6.04894474
Au       2.96686375       0.00000000      -6.04894474
Au       5.93372749       0.00000000      -6.04894474
Au       0.00000000       2.96686375      -6.04894474
Au       2.96686375       2.96686375      -6.04894474
Au       5.93372749       2.96686375      -6.04894474
Au       0.00000000       5.93372749      -6.04894474
Au       2.96686375       5.93372749      -6.04894474
Au       5.93372749       5.93372749      -6.04894474
Au       1.48331796       1.48374754      -3.97861230
Au       4.45036882       1.48364687      -3.97803820
Au       7.41728535       1.48372246      -3.97841883
Au       1.48330486       4.45114227      -3.98073112
Au       4.45028823       4.45111687      -3.97993258
Au       7.41740105       4.45106995      -3.98030116
Au       1.48336717       7.41599848      -3.98004311
Au       4.45025708       7.41614227      -3.97979650
Au       7.41721353       7.41609459      -3.97995768
Au      -0.00013057      -0.00062908      -1.88823630
Au       2.96703020      -0.00068489      -1.88835008
Au       5.93366883      -0.00057767      -1.88842611
Au      -0.00018418       2.96774828      -1.88847354
Au       2.96706111       2.96774416      -1.88850830
Au       5.93370922       2.96766318      -1.88842101
Au      -0.00012609       5.93348834      -1.88909417
Au       2.96701204       5.93355094      -1.88905213
Au       5.93369837       5.93347805      -1.88896816
```

Adsorbate@slab

CO@Au(111) ontop

```
38
Lattice="8.900591307799893 0.0 0.0 4.450295653899946 7.708134587337402 0.0 0.0 0.0 47.267302187375954"
Properties=species:S:1:pos:R:3 pbc="T T T"
Au       0.00000000       0.00000000      -8.63365116
Au       2.96686374       0.00000000      -8.63365116
Au       5.93372749       0.00000000      -8.63365116
Au       1.48343187       2.56937937      -8.63365116
Au       4.45029562       2.56937937      -8.63365116
Au       7.41715936       2.56937937      -8.63365116
Au       2.96686374       5.13875874      -8.63365116
Au       5.93372749       5.13875874      -8.63365116
Au       8.90059123       5.13875874      -8.63365116
Au       1.48343187       0.85645979      -6.21121705
Au       4.45029562       0.85645979      -6.21121705
Au       7.41715936       0.85645979      -6.21121705
Au       2.96686374       3.42583916      -6.21121705
Au       5.93372749       3.42583916      -6.21121705
Au       8.90059123       3.42583916      -6.21121705
Au       4.45029562       5.99521854      -6.21121705
Au       7.41715936       5.99521854      -6.21121705
Au      10.38402311       5.99521854      -6.21121705
Au      -0.00000215       1.70133207      -3.85857465
Au       2.98797653       1.72511970      -3.78438347
Au       5.91261714       1.72511787      -3.78438820
Au       1.48149266       4.28342342      -3.84384387
Au       4.45029787       4.25791273      -3.78438492
Au       7.41909658       4.28342105      -3.84385171
Au       2.97689564       6.85747698      -3.85854744
Au       5.92369273       6.85747239      -3.85855363
Au       8.90059171       6.84943816      -3.84385457
Au       0.00000889      -0.00000533      -1.40437976
Au       2.95133205      -0.04138120      -1.42382358
Au       5.94926384      -0.04137992      -1.42382121
Au       1.43981351       2.57661382      -1.42383278
Au       4.45030157       2.56938831      -1.34405389
Au       7.46078901       2.57661047      -1.42382649
Au       2.93879367       5.17291969      -1.42383316
Au       5.96179561       5.17291638      -1.42382355
Au       8.90058201       5.13876839      -1.39191762
C        4.45029647       2.56937966       0.66237477
O        4.45029838       2.56936438       1.81103885
```

CO@Au(111) bridge

```
38
Lattice="8.900591307799893 0.0 0.0 4.450295653899946 7.708134587337402 0.0 0.0 0.0 47.267302187375954"
Properties=species:S:1:pos:R:3 pbc="T T T"
Au       0.00000000       0.00000000      -8.63365116
Au       2.96686374       0.00000000      -8.63365116
Au       5.93372749       0.00000000      -8.63365116
Au       1.48343187       2.56937937      -8.63365116
Au       4.45029562       2.56937937      -8.63365116
Au       7.41715936       2.56937937      -8.63365116
Au       2.96686374       5.13875874      -8.63365116
Au       5.93372749       5.13875874      -8.63365116
Au       8.90059123       5.13875874      -8.63365116
Au       1.48343187       0.85645979      -6.21121705
Au       4.45029562       0.85645979      -6.21121705
Au       7.41715936       0.85645979      -6.21121705
Au       2.96686374       3.42583916      -6.21121705
Au       5.93372749       3.42583916      -6.21121705
Au       8.90059123       3.42583916      -6.21121705
Au       4.45029562       5.99521854      -6.21121705
Au       7.41715936       5.99521854      -6.21121705
Au      10.38402311       5.99521854      -6.21121705
Au      -0.03326887       1.74384399      -3.81783962
Au       3.00013118       1.74383670      -3.81784147
Au       5.93372920       1.72269636      -3.76883612
Au       1.48343319       4.30429614      -3.85317233
Au       4.46805345       4.25974732      -3.80966564
Au       7.39940597       4.25974875      -3.80965784
Au       2.98228690       6.85662664      -3.85123186
Au       5.93372991       6.88856775      -3.86530260
Au       8.88516433       6.85662309      -3.85121784
Au      -0.00438440      -0.00050179      -1.40507410
Au       2.97123911      -0.00050529      -1.40507448
Au       5.93372715      -0.12014507      -1.46604497
Au       1.48343307       2.57057521      -1.43769141
Au       4.32708685       2.54498242      -1.23035025
Au       7.54036028       2.54497849      -1.23035220
Au       2.98266266       5.12486407      -1.41684486
Au       5.93372687       5.24951661      -1.45450984
Au       8.88479967       5.12486007      -1.41685195
C        5.93372362       2.58842020       0.14379152
O        5.93372126       2.59439790       1.32022925
```

CO@Au(111) fcc

```
38
Lattice="8.900591307799893 0.0 0.0 4.450295653899946 7.708134587337402 0.0 0.0 0.0 47.267302187375954"
Properties=species:S:1:pos:R:3 pbc="T T T"
Au       0.00000000       0.00000000      -8.63365116
Au       2.96686374       0.00000000      -8.63365116
Au       5.93372749       0.00000000      -8.63365116
Au       1.48343187       2.56937937      -8.63365116
Au       4.45029562       2.56937937      -8.63365116
Au       7.41715936       2.56937937      -8.63365116
Au       2.96686374       5.13875874      -8.63365116
Au       5.93372749       5.13875874      -8.63365116
Au       8.90059123       5.13875874      -8.63365116
Au       1.48343187       0.85645979      -6.21121705
Au       4.45029562       0.85645979      -6.21121705
Au       7.41715936       0.85645979      -6.21121705
Au       2.96686374       3.42583916      -6.21121705
Au       5.93372749       3.42583916      -6.21121705
Au       8.90059123       3.42583916      -6.21121705
Au       4.45029562       5.99521854      -6.21121705
Au       7.41715936       5.99521854      -6.21121705
Au      10.38402311       5.99521854      -6.21121705
Au      -0.02323799       1.72633025      -3.81653084
Au       2.99016521       1.72634455      -3.81644560
Au       5.93368625       1.73878653      -3.78791819
Au       1.48344925       4.28206829      -3.83684905
Au       4.47272700       4.26934225      -3.78794877
Au       7.39480297       4.26944393      -3.78800816
Au       2.96703853       6.85177623      -3.83682670
Au       5.93374342       6.82485868      -3.81651096
Au       8.90044961       6.85177908      -3.83686376
Au      -0.00028975      -0.00014920      -1.39346525
Au       2.96714636      -0.00023816      -1.39349083
Au       5.93370248      -0.01206733      -1.41652860
Au       1.48335600       2.56968885      -1.39345674
Au       4.34588696       2.50914051      -1.24921619
Au       7.52132676       2.50924555      -1.24958307
Au       2.95632269       5.14483395      -1.41653475
Au       5.93369688       5.25909012      -1.24952703
Au       8.91110987       5.14484608      -1.41652444
C        5.93361598       3.42577942      -0.01878416
O        5.93381421       3.42583845       1.16857492
```

CO@Au(100) ontop

```
38
Lattice="8.900591307799893 0.0 0.0 0.0 8.900591307799893 0.0 0.0 0.0 46.29366650421573"
Properties=species:S:1:pos:R:3 pbc="T T T"
Au       1.48343187       1.48343187      -8.14683421
Au       4.45029562       1.48343187      -8.14683421
Au       7.41715936       1.48343187      -8.14683421
Au       1.48343187       4.45029562      -8.14683421
Au       4.45029562       4.45029562      -8.14683421
Au       7.41715936       4.45029562      -8.14683421
Au       1.48343187       7.41715936      -8.14683421
Au       4.45029562       7.41715936      -8.14683421
Au       7.41715936       7.41715936      -8.14683421
Au       0.00000000       0.00000000      -6.04894474
Au       2.96686374       0.00000000      -6.04894474
Au       5.93372749       0.00000000      -6.04894474
Au       0.00000000       2.96686374      -6.04894474
Au       2.96686374       2.96686374      -6.04894474
Au       5.93372749       2.96686374      -6.04894474
Au       0.00000000       5.93372749      -6.04894474
Au       2.96686374       5.93372749      -6.04894474
Au       5.93372749       5.93372749      -6.04894474
Au       1.51522097       1.51522020      -3.93299908
Au       4.41850422       1.51522405      -3.93299605
Au       7.41716009       1.47331650      -4.01076777
Au       1.51522351       4.41850445      -3.93299462
Au       4.41850619       4.41850669      -3.93299921
Au       7.41715810       4.46041064      -4.01076876
Au       1.47332070       7.41715757      -4.01076866
Au       4.46040697       7.41716142      -4.01076819
Au       7.41716056       7.41715861      -3.97725704
Au       0.00150223       0.00150051      -1.90783320
Au       2.96686342      -0.05644649      -1.89101935
Au       5.93222565       0.00150014      -1.90783651
Au      -0.05643731       2.96686139      -1.89100927
Au       2.96687248       2.96685337      -1.71979930
Au       5.99017085       2.96686585      -1.89100760
Au       0.00150186       5.93222648      -1.90783720
Au       2.96686394       5.99016724      -1.89101961
Au       5.93222408       5.93222899      -1.90783201
C        2.96685934       2.96686609       0.26010414
O        2.96686232       2.96686305       1.41047595
```

CO@Au(100) bridge

```
38
Lattice="8.900591307799893 0.0 0.0 0.0 8.900591307799893 0.0 0.0 0.0 46.29366650421573"
Properties=species:S:1:pos:R:3 pbc="T T T"
Au       1.48343187       1.48343187      -8.14683421
Au       4.45029562       1.48343187      -8.14683421
Au       7.41715936       1.48343187      -8.14683421
Au       1.48343187       4.45029562      -8.14683421
Au       4.45029562       4.45029562      -8.14683421
Au       7.41715936       4.45029562      -8.14683421
Au       1.48343187       7.41715936      -8.14683421
Au       4.45029562       7.41715936      -8.14683421
Au       7.41715936       7.41715936      -8.14683421
Au       0.00000000       0.00000000      -6.04894474
Au       2.96686375       0.00000000      -6.04894474
Au       5.93372749       0.00000000      -6.04894474
Au       0.00000000       2.96686375      -6.04894474
Au       2.96686375       2.96686375      -6.04894474
Au       5.93372749       2.96686375      -6.04894474
Au       0.00000000       5.93372749      -6.04894474
Au       2.96686375       5.93372749      -6.04894474
Au       5.93372749       5.93372749      -6.04894474
Au       1.50577270       1.49801904      -3.96470983
Au       4.45029745       1.51155687      -3.96471491
Au       7.39480949       1.49802245      -3.96470163
Au       1.50578238       4.43570713      -3.96470736
Au       4.45030193       4.42217055      -3.96471468
Au       7.39481982       4.43570588      -3.96470624
Au       1.47487510       7.41715865      -3.99969086
Au       4.45029361       7.41716184      -3.97887772
Au       7.42571255       7.41716050      -3.99968956
Au       0.00000285      -0.01296608      -1.88473093
Au       2.95566615      -0.02345265      -1.90342318
Au       5.94492874      -0.02345106      -1.90341913
Au       0.00000781       2.96686434      -1.86969895
Au       2.81450891       2.96686659      -1.71332823
Au       6.08610379       2.96685984      -1.71330214
Au      -0.00000767       5.94669532      -1.88473052
Au       2.95566103       5.95718330      -1.90342299
Au       5.94492070       5.95717973      -1.90342675
C        4.45029818       2.96685808      -0.37427605
O        4.45028132       2.96685398       0.80270232
```

CO@Au(100) hollow

```
38
Lattice="8.900591307799893 0.0 0.0 0.0 8.900591307799893 0.0 0.0 0.0 46.29366650421573"
Properties=species:S:1:pos:R:3 pbc="T T T"
Au       1.48343187       1.48343187      -8.14683421
Au       4.45029562       1.48343187      -8.14683421
Au       7.41715936       1.48343187      -8.14683421
Au       1.48343187       4.45029562      -8.14683421
Au       4.45029562       4.45029562      -8.14683421
Au       7.41715936       4.45029562      -8.14683421
Au       1.48343187       7.41715936      -8.14683421
Au       4.45029562       7.41715936      -8.14683421
Au       7.41715936       7.41715936      -8.14683421
Au       0.00000000       0.00000000      -6.04894474
Au       2.96686374       0.00000000      -6.04894474
Au       5.93372749       0.00000000      -6.04894474
Au       0.00000000       2.96686374      -6.04894474
Au       2.96686374       2.96686374      -6.04894474
Au       5.93372749       2.96686374      -6.04894474
Au       0.00000000       5.93372749      -6.04894474
Au       2.96686374       5.93372749      -6.04894474
Au       5.93372749       5.93372749      -6.04894474
Au       1.50495761       1.50495066      -3.97316925
Au       4.45030203       1.51381768      -3.96015525
Au       7.39564683       1.50494529      -3.97318495
Au       1.51381796       4.45030378      -3.96014898
Au       4.45029867       4.45029717      -4.04997416
Au       7.38677726       4.45029225      -3.96014741
Au       1.50494701       7.39564560      -3.97318202
Au       4.45029491       7.38677555      -3.96015094
Au       7.39564212       7.39564180      -3.97317229
Au      -0.00000164      -0.00000094      -1.92919778
Au       2.97507041       0.00000184      -1.89579297
Au       5.92552200       0.00000118      -1.89579889
Au       0.00000927       2.97506257      -1.89579518
Au       2.93764445       2.93763512      -1.79487527
Au       5.96294209       2.93765051      -1.79489243
Au       0.00000003       5.92553211      -1.89579457
Au       2.93766155       5.96294661      -1.79489116
Au       5.96295705       5.96296138      -1.79490098
C        4.45027751       4.45025876      -0.60974687
O        4.45029715       4.45028112       0.57478823
```

References

[1] Jun Haruyama, Tamio Ikeshoji, and Minoru Otani. Electrode potential from density functional theory calculations combined with implicit solvation theory. *Phys. Rev. Mater.*, 2(9):095801, September 2018.

[2] John J. Karnes, Stephen E. Weitzner, Sneha A. Akhade, Sarah E. Baker, Eric B. Duoss, and Joel B. Varley. A Hybrid Quantum–Classical Study of Ion Adsorption at the Copper Electrode. *J. Phys. Chem. C*, 126(30):12413–12423, August 2022.
